# Structure–Property Relationships of Hot-Pressed Wood–Polymer Composite Boards from Recycled ABS Edge-Banding Waste and Wood Fibers

**DOI:** 10.3390/polym18131591

**Published:** 2026-06-26

**Authors:** Viktor Savov, Petar Antov, Alexandrina Kostadinova-Slaveva, Ekaterina Todorova, Matei Botev, Georgi Ivanov, Viktoria Dudeva, Martina Todorova, Konstantinos Ninikas, Stoyko Petrin, Anton Kuzmin

**Affiliations:** 1 Faculty of Forest Industry, University of Forestry, 1797 Sofia, Bulgaria; p.antov@ltu.bg (P.A.); mmbotev017@ltu.bg (M.B.); georgi_ivanov@ltu.bg (G.I.); v.dudeva@ltu.bg (V.D.); martinatodorova@ltu.bg (M.T.); 2 Center of Competence “Clean Technologies for a Sustainable Environment—Water, Waste, Energy for a Circular Economy”, 1407 Sofia, Bulgaria; aslaveva@ltu.bg (A.K.-S.); etodorova@ltu.bg (E.T.); 3 Faculty of Ecology and Landscape Architecture, University of Forestry, 1797 Sofia, Bulgaria; 4 Department of Forestry, Wood Sciences & Design, School of Technology, University of Thessaly, 43100 Karditsa, Greece; kninikas@uth.gr; 5 Faculty of Chemical Technologies, University of Chemical Technology and Metallurgy, 1757 Sofia, Bulgaria; stpetrin@uctm.edu; 6 Laboratory of Advanced Composite Materials and Technologies, Federal State Budgetary Educational Institution of Higher Education, Plekhanov Russian University of Economics, 117997 Moscow, Russia; kuzmin.a.m@yandex.ru

**Keywords:** wood–polymer composites, recycled ABS, edge-banding waste, wood fibers, thermoplastic matrix, composite boards, structural homogeneity

## Abstract

Recycled thermoplastics offer a promising route for valorizing industrial residues and developing thermoplastic-bonded wood-based panels without added formaldehyde-based resins. In this study, experimental wood–polymer composite boards were produced from recycled acrylonitrile–butadiene–styrene (ABS) edge-banding waste used as the polymer matrix and industrial wood fibers used as the lignocellulosic reinforcement. The boards were manufactured at target densities of 800–1200 kg·m^−3^ and wood fiber contents of 10–30%, followed by the evaluation of selected physical and mechanical properties, including water absorption, thickness swelling, modulus of elasticity and bending strength. Thermogravimetric analysis of the recycled ABS edge-banding material and qualitative optical microscopy of the board surfaces were used to support, but not independently prove, the interpretation of the composite structure. The recycled ABS waste enabled the formation of compact boards, with density exerting the strongest influence on water resistance and bending performance. The regression models indicated a balanced region at 21.84 wt.% wood fibers and 1134 kg·m^−3^, corresponding to predicted water absorption of 1.62%, thickness swelling of 3.22%, modulus of elasticity of 2931 N·mm^−2^ and bending strength of 22.20 N·mm^−2^. Optical microscopy suggested a more continuous ABS-rich surface in the most homogeneous specimens, whereas local accumulations of fine particles and areas of limited polymer coverage were observed on the opposite surface. These findings demonstrate the potential of recycled ABS edge-banding waste for wood–polymer board production, while indicating that additional feedstock cleaning and sieving should be investigated in subsequent work to improve furnish uniformity and structural homogeneity.

## 1. Introduction

Wood-based panels are among the most important engineered materials used in furniture manufacturing, interior products and construction because they enable efficient utilization of lignocellulosic raw materials and provide boards with controlled dimensions and predictable performance. Conventional particleboards and fiberboards are commonly bonded with formaldehyde-based thermosetting resins, mainly urea–formaldehyde, phenol–formaldehyde and melamine–urea–formaldehyde systems. Although these adhesives are technologically mature and economically efficient, their use is associated with formaldehyde emissions during manufacture and service life. For this reason, the development of alternative bonding concepts and formaldehyde-free panel materials remains an important research direction in wood-based composite technology [1].

One promising approach is the use of thermoplastic polymers as matrix or bonding phases in wood-based panels. During hot pressing, thermoplastics soften or melt, penetrate the voids between wood particles or fibers and subsequently solidify during cooling, forming a consolidated wood–polymer structure. Compared with conventional adhesive-bonded panels, thermoplastic-bonded wood-based materials may offer improved water resistance, good processability and the possibility of incorporating recycled plastics as secondary raw materials [1,2]. Flat-pressed wood–polymer composites are particularly relevant to the wood-based panel industry, because their production is closer to conventional particleboard and fiberboard technologies than extrusion or injection molding [3,4]. Previous studies have shown that flat-pressed WPC panels can be produced with different thermoplastic matrices and wood fractions; however, their final performance depends strongly on polymer type, wood-particle or fiber geometry, matrix-to-wood ratio, mat consolidation, moisture exposure and machining behavior [5,6,7,8].

The use of recycled plastics in wood–polymer composites is strongly aligned with circular economy principles, because it enables polymer-containing industrial residues to be converted into durable composite products rather than being directed to low-value recovery routes or disposal. This is especially relevant for pre-consumer plastic waste streams generated in furniture manufacturing, where relatively concentrated residues may still retain useful thermoplastic properties but are not always suitable for direct reprocessing into high-grade polymer products. In this context, WPCs provide a practical material-recovery pathway by combining recycled thermoplastics with lignocellulosic materials to reduce the demand for virgin fossil-based polymers, extend the service life of plastic residues and create value-added panel products from secondary raw materials. Studies on WPCs produced from waste electrical and electronic equipment, construction and demolition waste and other recycled polymer sources have demonstrated that recycled plastics can be successfully used as matrix phases, although their variable composition, possible contamination and differences in melt behavior must be carefully considered [9,10,11,12]. This is consistent with the broader WPC literature, where recycled polymer quality, fiber characteristics, particle-size distribution, matrix–fiber compatibility and processing route are repeatedly identified as key factors controlling physical and mechanical performance [13,14,15,16,17,18]. These issues are particularly important for recycled edge-banding waste, which may contain pigments, additives, residual wood dust or particles from previous machining operations. Such heterogeneity can influence polymer flow, fiber encapsulation, interfacial bonding and the structural homogeneity of the resulting composite boards.

Acrylonitrile–butadiene–styrene (ABS) is a technically attractive thermoplastic matrix for wood–polymer composites because of its relatively high stiffness, impact resistance, dimensional stability and good processability. Matuana et al. demonstrated that ABS can be processed with wood flour using conventional polymer-processing equipment and that ABS-based wood–plastic composites can exhibit favorable stiffness compared with composites based on some commodity thermoplastics [19]. Yeh et al. further showed that both virgin and recovered ABS can be used in WPC formulations containing 50 wt.% wood flour, and that the mechanical behavior of the resulting composites is influenced not only by the origin of the ABS, but also by the wood fraction, dispersion quality and interfacial compatibility [20]. Studies on ABS/wood sawdust composites have similarly confirmed that the lignocellulosic phase can enhance stiffness; however, insufficient dispersion, poor wetting and weak interfacial adhesion may reduce strength, elongation at break and impact resistance [21,22]. Therefore, the main technological challenge in ABS-based wood–polymer panels is not merely to replace a conventional adhesive with a thermoplastic matrix, but to ensure adequate polymer softening and flow, continuous matrix formation, effective fiber embedment and uniform composite structure during pressing.

Recycled ABS from furniture edge-banding operations represents a particularly relevant, but still insufficiently explored, secondary raw material for wood–polymer composite boards. ABS edge banding is widely used to finish the exposed edges of melamine-faced particleboards and other furniture panels. During trimming and leveling, excess edge-banding material is removed, generating ABS-rich shavings and particles as a pre-consumer industrial waste stream. Georgescu and Coşereanu investigated recycled ABS shavings from edge-banding operations in combination with recycled polyethylene and wood fibers for thermal insulating panels, showing that this waste stream can be incorporated into panel-type composite materials [23]. Recent studies have also confirmed that ABS can be combined with lignocellulosic particles such as pine or hemp fractions, and that preheating, fraction size and ABS content can influence compaction, homogenization and final material formation [24]. At the same time, studies on WPCs and recycled thermoplastic composites show that fiber size, particle-size distribution, reprocessing history and weathering or previous service exposure may strongly affect morphology, dimensional stability and mechanical properties [14,15,16,17,18]. However, most available studies have focused on extruded WPC profiles, sawdust-filled ABS composites, low-density insulating materials or thermal/acoustic applications. In contrast, denser hot-pressed board materials produced from recycled ABS edge-banding waste and intentionally added wood fibers remain less investigated. This gap is important because such materials are closer to conventional wood-based panel technology and may offer a practical route for converting furniture-industry ABS residues into value-added composite boards.

This aspect is central for the present study. The investigated formulation is based on recycled ABS edge-banding waste as the thermoplastic matrix and added wood fibers as the lignocellulosic phase. Fine wood-derived particles observed in the composite structure were therefore interpreted cautiously as likely residual fines associated with the preparation of the recycled edge-banding stream rather than as an intentionally designed third component. Because no separate FTIR, SEM-EDS or chemical quantification of these fines was performed in the present work, the related discussion is limited to their possible technological role in local furnish heterogeneity, polymer coverage and water-accessible surface area. Consequently, the preparation of the recycled ABS fraction, particularly sieving and removal of excessive dust-like material, is considered a practical processing issue that deserves dedicated investigation in follow-up studies.

The aim of this study was to evaluate hot-pressed wood–polymer composite boards produced from recycled ABS edge-banding waste and industrial wood fibers. The work combines the assessment of selected physical and mechanical properties, including density, 24 h water absorption, thickness swelling, modulus of elasticity and bending strength, with microscopic observations of the board surface structure. Particular attention is paid to the relationships between polymer continuity, wood fiber embedment, local structural heterogeneity and the presence of residual fine wood-derived particles in the recycled ABS fraction. In this way, this study contributes to the development of circular thermoplastic-bonded board materials based on furniture-industry polymer waste and produced without added formaldehyde-based resins. It also identifies a practical technological requirement for this waste stream: cleaning and sieving the ground ABS edge-banding waste before composite-board production in order to improve furnish uniformity and board homogeneity.

## 2. Materials and Methods

### 2.1. Raw Materials

Recycled acrylonitrile–butadiene–styrene (ABS) edge-banding waste and industrial wood fibers were used as the main raw materials for the production of laboratory wood–polymer composite boards. The ABS edge-banding waste was supplied by MAB Stil BG Ltd. (Sofia, Bulgaria) and originated as a secondary production waste stream generated during furniture edge-banding operations. The material consisted of ABS-based shavings and particles obtained after trimming and finishing of edge-banded wood-based panels. In the present study, the recycled ABS edge-banding waste was used as the thermoplastic matrix.

Before board production, the ABS edge-banding waste was mechanically size-reduced using a laboratory hammer mill developed as a prototype at the University of Forestry, Sofia, Bulgaria. The mill was operated in pass-through mode, without using an outlet screen. Therefore, the resulting particle-size distribution was not predetermined by a fixed sieve aperture during milling, but reflected the actual fragmentation behavior of the recycled ABS edge-banding waste under the applied milling conditions. This is important because such waste may contain not only ABS material, but also residual fine wood dust and small lignocellulosic particles originating from insufficiently cleaned edge-banding residues.

Industrial wood fibers supplied by Kronospan AD (Veliko Tarnovo, Bulgaria) were used as the lignocellulosic reinforcing/filling phase. The fibers represented an industrial raw material intended for the manufacture of wood fiberboards. No formaldehyde-based adhesive or additional thermosetting binder was used in the board formulation.

It should be noted that, in addition to the intentionally added industrial wood fibers, the recycled ABS fraction contained a certain amount of residual fine wood dust and small lignocellulosic particles. These fines were not added as a separate formulation component, but originated mainly from insufficiently cleaned ABS edge-banding residues after grinding. Their presence was therefore considered in the interpretation of the fractional composition of the recycled ABS waste and the microscopic structure of the produced composite boards.

### 2.2. Sieve Analysis of the Recycled ABS Edge-Banding Waste

The fractional composition of the ground recycled ABS edge-banding waste was determined by sieve analysis using a BA200N sieve analyzer (CISA, Barcelona, Spain). The retained fraction on each sieve was expressed as a percentage of the total sample mass. Since the hammer mill was operated without an outlet screen, the sieve analysis was used to characterize the actual particle-size distribution obtained after pass-through milling. The results of the fractional analysis were further used to support the interpretation of board structure, surface homogeneity and the observed accumulation of fine wood-derived particles.

### 2.3. Experimental Design

The experimental design was based on two variable factors: target board density and wood fiber content. The target density was varied at three levels: 800, 1000 and 1200 kg·m^−3^. The wood fiber content was varied from 10 to 30 wt.%, corresponding to recycled ABS contents of 90 to 70 wt.%. Thus, the total solid content of each formulation consisted only of industrial wood fibers and recycled ABS edge-banding waste. The nominal board dimensions were 400 mm × 400 mm × 10 mm. The experimental matrix is presented in Table 1.

### 2.4. Preparation of Composite Boards

For each board, the required amounts of industrial wood fibers and recycled ABS edge-banding waste were weighed according to the experimental design. The wood fibers were first placed in a mechanical mixer equipped with rotating blades and mixed for 3 min to loosen and homogenize the fiber mass. The required amount of recycled ABS waste was then added gradually in two portions in order to improve the distribution of the polymer particles within the fiber furnish. The mixed furnish was manually formed into a mat using a forming frame placed on aluminum caul plates previously treated with a release agent. After mat formation, preliminary cold consolidation was applied to improve mat stability. The forming frame was then removed, and the mat was placed between aluminum caul plates with thickness stops.

The boards were hot pressed using a laboratory press, type PMC ST 100 (Manni S.P.A., Verona, Italy). Hot pressing was carried out at 220 °C using a stepwise pressure regime. The selected temperature was intended to promote softening and flow of the recycled ABS phase, enabling improved contact between polymer particles and wood fibers, while remaining within a processing range in which intensive thermal degradation of the recycled ABS waste was not expected during the relatively short pressing cycle. The pressure was maintained at 3 MPa for 2 min to ensure initial mat compression, particle contact and heat transfer through the furnish. It was then reduced to 0.6 MPa for 4 min to maintain consolidation while allowing further polymer softening, redistribution and partial stress relaxation. In the final stage, the pressure was reduced to 0.2 MPa for 4 min in order to stabilize board thickness and limit excessive internal stress development before unloading. The total hot-pressing cycle was 10 min. After hot pressing, the boards were cooled in a cold press for 20 min under a pressure of 0.2 MPa, allowing the softened ABS matrix to solidify under restraint and reducing the risk of springback or thickness recovery after pressing. Before testing, all boards and test specimens were conditioned at 20 ± 2 °C and 65 ± 5% relative humidity until constant mass.

### 2.5. Preparation of Test Specimens

Test specimens were prepared according to the general principles of EN 326-1 for sampling, cutting and expression of test results for wood-based panels [25]. Six test specimens were used for each experimental condition and property.

Specimens for density, water absorption and thickness swelling had dimensions of 50 mm × 50 mm. Specimens for bending tests had a length of 250 mm and a width of 50 mm. The support span in the three-point bending test was 200 mm.

### 2.6. Density

The density of the boards was determined according to EN 323 [26]. Six specimens with dimensions of 50 mm × 50 mm were tested for each experimental series. The mass of each specimen was measured using an electronic balance Kern (KERN & SOHN GmbH, Balingen, Germany) with an accuracy of 0.01 g. The length and width were measured using a caliper, while thickness was measured using a thickness gauge. The density values were calculated in accordance with the standard procedure and expressed in kg·m^−3^.

### 2.7. Water Absorption and Thickness Swelling

Water absorption and thickness swelling were determined after 24 h immersion in water. Thickness swelling was evaluated according to EN 317 [27], while water absorption was determined gravimetrically using the same specimens and immersion procedure. Six specimens with dimensions of 50 mm × 50 mm were tested for each board series.

Before immersion, the mass and thickness of each specimen were measured. The specimens were then fully immersed in water at 20 ± 1 °C for 24 h, ensuring that they did not touch one another or the walls of the container. After immersion, excess surface water was removed, and the specimens were immediately reweighed and remeasured. The results were expressed as percentages.

### 2.8. Modulus of Elasticity and Bending Strength

The modulus of elasticity and bending strength were determined according to EN 310 [28] using a universal testing machine HST-MWW-50E (Jinan Hensgrand Instrument, Jinan, China). Six specimens were tested for each experimental series. The specimens had a length of 250 mm and a width of 50 mm, and the support span was 200 mm. The tests were performed under three-point bending conditions, and the modulus of elasticity and bending strength were calculated according to the standard procedure.

### 2.9. Microscopic Characterization

The surface structure of the composite boards was examined by optical microscopy at 32× magnification. Representative images were taken from both surfaces of the boards in order to describe the visible distribution of the ABS-rich phase, the apparent embedment of wood fibers, the presence of fine particles and agglomerates and the overall surface homogeneity of the composites. The method was used as a qualitative surface-characterization tool and was not intended to provide quantitative morphological analysis, fracture-surface information, elemental composition or direct evidence of interfacial adhesion.

Particular attention was paid to areas with apparent polymer coverage and visible fiber incorporation, as well as to local zones with accumulations of fine material and limited polymer coverage. The microstructural observations were used only to support a cautious interpretation of the physical and mechanical test results. More detailed verification of matrix–fiber adhesion, cross-sectional morphology and the chemical nature of the fine fraction will require SEM, SEM-EDS, FTIR and related analyses in future work.

### 2.10. Thermogravimetric Analysis

Thermogravimetric analysis of the recycled ABS edge-banding waste was performed using an HS-TGA-103 thermogravimetric analyzer (Yantai Stark Instrument, Yantai, China). The sample mass was 24.48 mg. The analysis was carried out in air using open crucibles. The exported measurement record covered the temperature interval from approximately 69 °C to 398 °C, at a heating rate of 10 °C min^−1^. TG and DTG curves were recorded to evaluate mass-loss behavior under oxidative conditions, with particular emphasis on whether an abrupt degradation event occurred near the selected hot-pressing temperature of 220 °C. Since TGA does not determine glass-transition temperature or softening behavior, the analysis was not used as a substitute for DSC. Instead, it was used as a processing-relevant stability check for the selected short pressing cycle. Because the run ended at approximately 400 °C and a substantial residual mass remained, the analysis was used primarily to assess thermal behavior within and above the processing-temperature range, not to describe the complete degradation profile of ABS.

### 2.11. Statistical and Regression Analysis

The experimental results were statistically processed using the specialized software QStatLab 6.0. The arithmetic mean, standard deviation and coefficient of variation were calculated for each experimental series and property.

The effects of board density and wood fiber content on the selected physical and mechanical properties were described using second-order regression models. Both coded-factor interpretation and the final uncoded equations were considered. In the manuscript, x_1_ denotes wood fiber content in wt.%, and x_2_ denotes target board density in kg·m^−3^ when the uncoded equations are reported.

$$\hat{Y}=b_{0}+b_{1}X_{1}+b_{2}X_{2}+b_{12}X_{1}X_{2}+b_{11}X_{1}^{2}+b_{22}X_{2}^{2}$$

where Y is the predicted response; x_1_ is the wood fiber content, wt.%; x_2_ is the board density, kg·m^−3^; and b_0_, b_1_, b_2_, b_11_, b_22_ and b_12_ are regression coefficients. In the case of thickness swelling, the non-significant interaction term was excluded from the final model.

Separate models were developed for water absorption, thickness swelling, modulus of elasticity and bending strength. Model adequacy was assessed using ANOVA, the coefficient of determination R^2^ and adjusted R^2^. The models were used for trend interpretation and for selecting rational factor regions within the investigated experimental domain. They were not treated as externally validated optimization models because no additional verification boards were produced at the predicted optima.

## 3. Results and Discussion

### 3.1. Fractional Composition of the Recycled ABS Edge-Banding Waste

**Figure 1:**
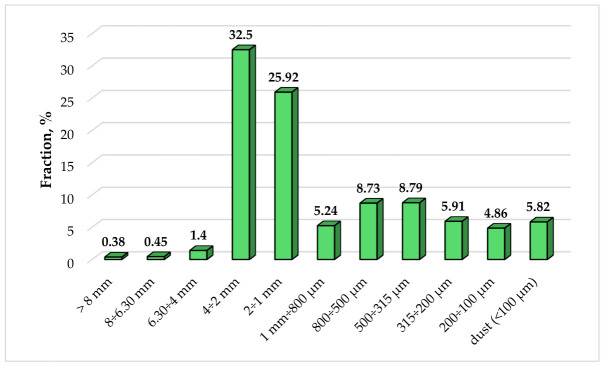
Fractional composition of the recycled ABS edge-banding waste.

The fractional composition of the recycled ABS edge-banding waste after pass-through milling is presented in Figure 1. Since the laboratory hammer mill was operated without an outlet screen, the obtained particle-size distribution was not controlled by a fixed sieve aperture during milling. Instead, it reflected the actual fragmentation behavior of the recycled ABS edge-banding waste under the applied milling conditions. Therefore, the sieve analysis was used to characterize the as-ground material and to evaluate the presence of coarse particles, fine fractions and dust that could influence mat formation, polymer distribution and board homogeneity.

The results show a clear predominance of medium-sized particles, especially the fractions retained on the 2 mm and 1 mm sieves, which accounted for 32.50% and 25.92%, respectively. Together, these two fractions represented 58.42% of the total mass. This indicates that the main part of the recycled ABS edge-banding waste was concentrated within a relatively narrow and technologically useful particle-size range. Such a distribution may be favorable for flat mat formation and hot pressing, because medium-sized particles can provide sufficient contact area while still maintaining adequate permeability and void spaces for heat transfer and polymer softening. In thermoplastic-bonded boards, particle-size distribution is a key structural parameter, since it influences furnish packing, the number and quality of interparticle contacts, heat transfer through the mat, polymer flow, void filling and the formation of a continuous matrix phase during pressing [1,3,4]. A predominance of excessively coarse particles could lead to local voids, poor surface quality and non-uniform consolidation, whereas an excessive amount of very fine material could increase the specific surface area and require more effective polymer flow for complete encapsulation. Therefore, the relatively high proportion of 1–2 mm particles suggests that the pass-through milling process produced a main ABS fraction suitable for laboratory board manufacture. Previous studies on WPCs have also demonstrated that fiber or particle size and processing method significantly affect composite morphology, water absorption, thickness swelling and mechanical performance [5,6,15,16].

The share of particles below 1 mm, including the 800 μm, 500 μm, 315 μm, 200 μm, 100 μm and dust fractions, was 39.35%. This relatively high amount of fine material can have a dual effect on board formation and performance. On the one hand, fine particles may fill voids between coarser ABS particles, improve furnish packing efficiency and contribute to better consolidation during hot pressing. Such a void-filling effect can be favorable for reducing open porosity in wood–polymer systems, increasing the number of contact points between polymer particles and supporting the formation of denser, more compact board structures. In addition, finer ABS particles may soften more rapidly than coarser particles because of their higher specific surface area, which can promote local polymer flow and improve the continuity of the matrix phase during pressing. Chaudemanche et al. [15] showed that the size distribution of wood-flour particles can strongly affect the physical and mechanical behavior of industrial HDPE-based WPCs, confirming that particle-size distribution should not be regarded only as a preparatory parameter, but as a structural factor influencing the final composite performance.

On the other hand, very fine particles increase the specific surface area of the furnish and therefore may require more effective polymer softening, flow and redistribution to ensure sufficient coverage and bonding. This effect becomes particularly critical if a part of the fine fraction is lignocellulosic rather than ABS-based, because hydrophilic fines may locally disturb the polymer-to-fiber ratio. Since the chemical composition of the fine fraction was not quantified in the present study, this interpretation is presented as a plausible technological explanation rather than as a directly proven mechanism. Delviawan et al. [16] reported that reducing wood particle size can improve mechanical properties only up to an optimum level, after which further size reduction may promote aggregation and reduce composite performance.

This interpretation is important because recycled ABS edge-banding waste differs from virgin polymer granules or controlled wood-flour fractions commonly used in many WPC studies. Edge-banding waste is a technologically heterogeneous secondary raw material and may contain pigments, additives, surface residues and fine lignocellulosic material originating from machining and trimming of edge-banded wood-based panels [9,10,23]. Therefore, the dust fraction of 5.82% should be considered a potential source of local heterogeneity, although its exact composition was not determined in this work. Similar concerns have been reported in studies on recycled plastics in WPCs, where the sorting, cleanliness and consistency of the recycled polymer fraction were shown to be decisive for composite performance [9,10].

The amount of coarse material was low, with the 4 mm and coarser fractions representing only 2.23% of the total mass. This is a favorable characteristic for board manufacture, since excessive coarse particles may create local voids, reduce the number of effective interparticle contact points and cause non-uniform heat transfer during pressing. In hot-pressed wood–polymer panels, such local heterogeneity can hinder polymer softening, flow and coalescence, thereby limiting the development of a continuous thermoplastic bonding phase [1,3,4]. The low proportion of coarse particles therefore indicates that pass-through milling was generally effective in reducing the ABS edge-banding waste to a particle-size range suitable for laboratory board production, while avoiding the formation of large fragments that could compromise mat consolidation and surface quality.

However, the relatively high proportion of fine and dust fractions also indicates a technological limitation of the as-ground recycled ABS material. Although fine particles may improve compactability and void filling, their uncontrolled accumulation can reduce surface uniformity, particularly if part of the fine fraction is lignocellulosic. For ABS-based wood–polymer composites, adequate polymer continuity and effective contact with the wood phase are important for mechanical performance [19,20,21,22]. Therefore, the fractional composition observed in the present study can be considered suitable for board formation, but not fully optimized without additional control of the fine fraction.

From a technological point of view, these results support the recommendation that an additional sieving or cleaning step should be evaluated after grinding of the ABS edge-banding waste. The objective should not necessarily be to remove all fine particles, because some fines can contribute to void filling and compaction, but rather to reduce excessive dust-like material and improve particle-size uniformity. Such processing improvements are expected to reduce local fine-particle accumulations and promote a more homogeneous ABS-rich structure during hot pressing. This conclusion is supported by the subsequent optical observations, while quantitative confirmation requires dedicated follow-up work on feedstock composition and board morphology.

### 3.2. Thermal Behavior of the Recycled ABS Edge-Banding Waste

**Figure 2:**
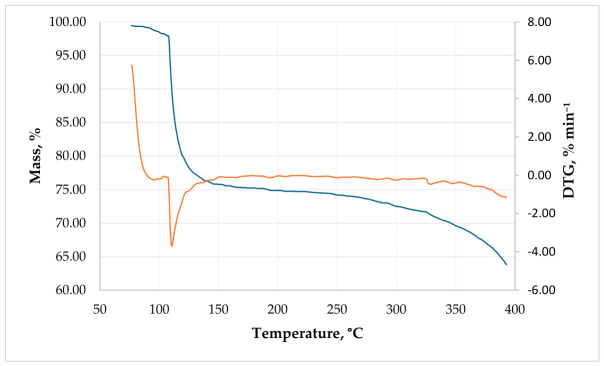
TG and DTG curves of the recycled ABS edge-banding waste measured in air at a heating rate of 10 °C min^−1^.

Figure 2 presents the TG and DTG curves of the recycled ABS edge-banding waste. The thermogravimetric profile indicates that the material retained a substantial part of its mass within the temperature range relevant to hot pressing, which supports the use of 220 °C as a processing temperature for the short laboratory pressing cycle. This temperature was selected to provide sufficient softening and flow of the ABS-rich phase while avoiding an abrupt mass-loss event according to the available TG/DTG data. However, because TGA does not identify glass transition or softening transitions, the selected temperature should be understood as a technologically justified processing temperature rather than as an optimum derived from thermal-transition analysis. DSC-based determination of the processing window is therefore proposed for subsequent studies.

The TG curve shows an initial mass decrease in the lower-temperature region, accompanied by a distinct DTG response. This event should be interpreted with caution and should not be directly attributed to the main thermal degradation of ABS. Previous studies have shown that the degradation behavior of ABS depends strongly on the testing atmosphere, heating rate, material origin and formulation, with the main degradation of neat or relatively clean ABS generally occurring at substantially higher temperatures than the initial low-temperature event observed here [29,30,31]. In the present recycled edge-banding waste, this low-temperature mass change may be related to residual moisture, volatile processing residues, additives, adhesive traces or fine lignocellulosic material [32,33]; however, no direct compositional analysis was performed to distinguish these contributions. Therefore, the explanation is presented as a cautious interpretation of a heterogeneous recycled feedstock rather than a confirmed assignment.

After this initial stage, the mass-loss rate becomes considerably lower over a broad intermediate temperature region. From a processing point of view, the absence of an abrupt mass-loss event near 220 °C suggests that the recycled ABS waste can be used under the selected short hot-pressing cycle without intensive mass loss. The TG/DTG results therefore support, but do not by themselves fully define, the selected pressing temperature. Additional DSC measurements would be needed to determine the glass-transition and softening behavior of this specific recycled ABS stream.

At higher temperatures, the TG curve shows a gradual increase in mass loss, while the DTG signal indicates the onset of more pronounced thermo-oxidative processes. Previous studies on ABS and recycled ABS-based materials have reported that degradation under oxidative conditions may occur over a broad temperature interval and may be affected by the butadiene phase, additives, previous processing history and the presence of impurities [29,30,31]. Compared with neat ABS, recycled ABS-containing waste streams may therefore exhibit earlier or broader mass-loss events. In the present case, this behavior is particularly relevant because the edge-banding waste was not a purified polymer fraction, but a real secondary industrial stream containing ABS particles together with fine residues from furniture-panel edge finishing.

It should also be noted that the TGA experiment was carried out dynamically in air using an open crucible, whereas board production was performed under hot-pressing conditions in a compacted mat and for a limited pressing time. Therefore, the TG/DTG results should not be interpreted as a direct simulation of the thermal conditions during pressing. The compacted mat, restricted oxygen access, heat-transfer gradients and short pressing cycle may all influence the actual thermal exposure of the ABS phase during board manufacture. The TGA results provide supporting evidence that the selected processing temperature lies within a thermally acceptable range for the recycled ABS edge-banding waste, while establishing a full thermal-processing window will require DSC and complementary analyses in future work.

### 3.3. Microscopic Appearance of the Board Surfaces

**Figure 3:**
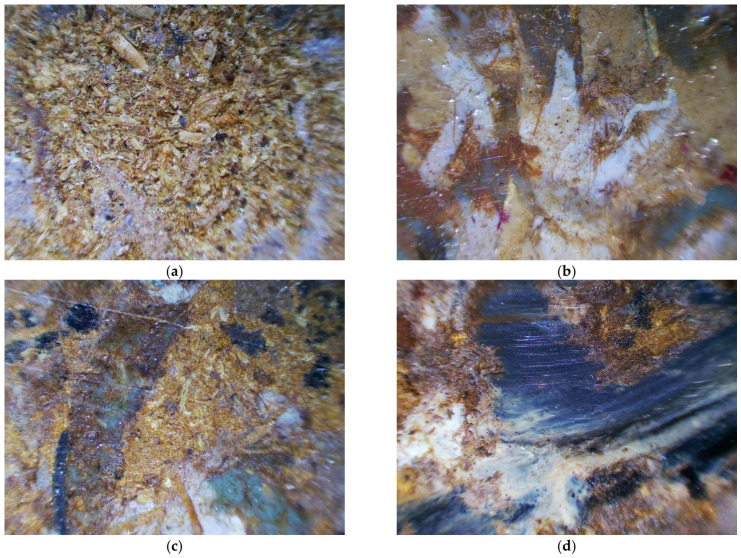

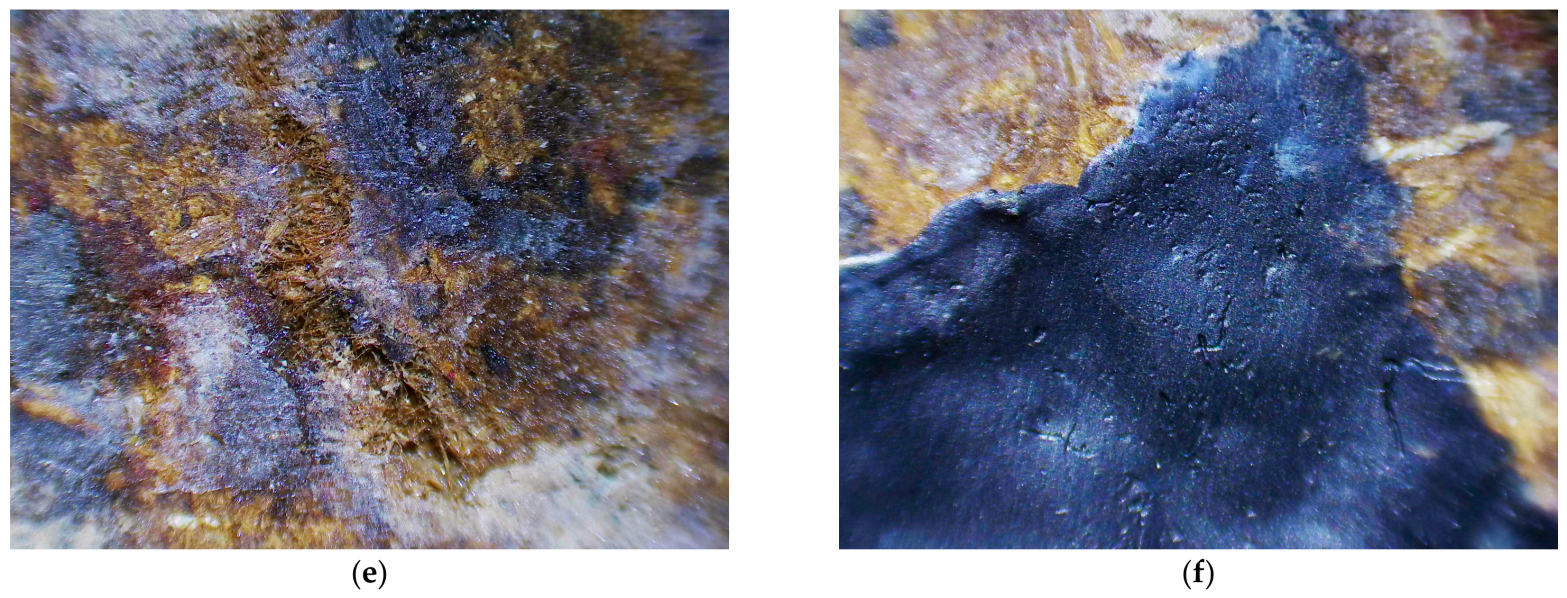
Representative optical micrographs of selected wood–polymer composite panels: (**a**) panel 1, upper surface; (**b**) panel 1, lower surface; (**c**) panel 4, upper surface; (**d**) panel 4, lower surface; (**e**) panel 9, upper surface; and (**f**) panel 9, lower surface. The lower surface corresponds to the side where fine particles were more pronounced after mat formation and pressing. Magnification: 32×.

Figure 3 presents representative optical micrographs of the surface structure of the wood–polymer composite boards produced from recycled ABS edge-banding waste and wood fibers. The images were selected to illustrate visible differences between comparatively homogeneous surfaces and surfaces where local accumulations of fine particles were more pronounced. Since the observations were performed at 32× magnification, the analysis was limited to qualitative surface morphology, including apparent ABS-rich regions, visible distribution of wood fibers and fines, agglomerates and zones with limited polymer coverage. The images do not provide direct evidence of chemical composition, interfacial adhesion or cross-sectional morphology.

In general, the more homogeneous surfaces showed a comparatively continuous ABS-rich phase, in which the thermoplastic matrix appeared more uniformly distributed around the wood fibers. Such morphology is usually favorable for wood–polymer composites because it may reduce open pathways for water ingress and improve the continuity of the load-bearing structure [34,35,36,37]. In the present study, this interpretation is based on optical surface observations and should not be considered a direct measurement of wetting, adhesion or stress transfer. The most representative example of a homogeneous surface was observed for Board 9, where the ABS-rich phase appeared relatively continuous and the fine particles were more uniformly incorporated into the surface layer.

By contrast, some lower/bottom surfaces showed more visible heterogeneity, including local accumulations of fine particles and regions where the polymer-rich phase did not appear to fully cover the lignocellulosic material. The most pronounced example of this behavior was observed for Board 1. The origin of these fine particles was not chemically quantified; therefore, they are discussed as likely wood-derived or mixed fine residues associated with the recycled edge-banding stream. From a materials perspective, such locally exposed or weakly covered lignocellulosic regions may increase hydrophilic sites and provide more readily accessible water pathways [34,36,37,38].

The observed differences between the two panel surfaces can be related to the mat-forming and pressing process. During mat formation, finer particles may migrate or accumulate towards one side of the mat, particularly when a heterogeneous recycled feedstock is used. As a result, the bottom surface of some panels exhibited a higher local concentration of fine material and zones with reduced apparent polymer coverage. This indicates that, in addition to nominal composition and pressing regime, the cleanliness and particle-size uniformity of the recycled ABS fraction are important factors for achieving a homogeneous wood–polymer composite structure.

The microscopic observations support one of the key technological recommendations of this study: after mechanical grinding of ABS edge-banding waste, an additional sieving and cleaning step should be considered to reduce excessive dust-like material and very fine fractions. This would be expected to improve particle-size uniformity, reduce local fine-particle accumulations and promote a more homogeneous ABS-rich matrix distribution during hot pressing. Quantitative verification of this assumption, including SEM, image analysis and chemical characterization of the fine fraction, is planned for subsequent work.

### 3.4. Density of the Laboratory Panels

Before analyzing the response surfaces and regression models, the actual density of the laboratory panels was evaluated, since density was one of the main controlled technological factors in the experimental design. In flat-pressed wood–polymer composite panels, density strongly affects particle packing, void volume, matrix consolidation, water uptake, thickness swelling and bending performance [3,4,6,7,12,38,39,40,41]. Therefore, verification of the achieved density levels was necessary for the correct interpretation of the subsequent physical and mechanical results, and the corresponding variation statistics are presented in Table 2.

The measured mean densities ranged from 775.4 to 1231.7 kg·m^−3^, covering the intended experimental range from approximately 800 to 1200 kg·m^−3^. The deviations from the target density levels were relatively small, ranging from −3.08% to +2.64%. This confirms that the laboratory pressing procedure provided sufficient control over panel densification, despite the use of a heterogeneous recycled ABS edge-banding fraction as the thermoplastic matrix. The achieved density range also provides a reliable basis for evaluating the influence of density on moisture resistance and bending performance within the studied factor space.

The coefficients of variation were generally low to moderate, varying from 0.76% to 5.97%. The lowest variability was observed for the high-density panels, particularly panels 4 and 8, whereas somewhat higher variation was recorded for the lower-density panels, especially panels 1 and 7. This behavior can be attributed to the higher sensitivity of lower-density mats to local differences in particle packing, fiber distribution, void formation and springback after pressing. Overall, the density results confirmed the reliability of the experimental design and supported the subsequent interpretation of the effects of density and wood fiber content on water absorption, thickness swelling and bending properties.

### 3.5. Experimental–Statistical Modelling of the Panel Properties

The effects of wood fiber content and panel density on the selected physical and mechanical properties were evaluated using second-order regression models. The statistical analysis was based on the QStatLab output obtained for 14 observations, including the replicated center-point measurements. The final equations are reported in actual factor units, where x_1_ is the wood fiber content, wt.%, and x_2_ is the target density, kg·m^−3^. This form is useful for practical interpretation of the response surfaces within the investigated domain.

The developed models were used primarily for interpretation of trends and for identifying rational technological regions within the studied factor space. Because the models were fitted to a limited laboratory-scale design and no additional verification boards were produced at the calculated optimum points, the reported optima should be interpreted as predicted candidate regions rather than externally validated processing parameters.

As shown in Table 3, all four regression models were statistically significant by ANOVA at *p* < 0.05 and were therefore considered suitable for describing the main response trends within the investigated factor space. The bending strength model showed the highest explanatory ability (R^2^ = 0.91591; R^2^ adj = 0.86335), followed by the modulus of elasticity model (R^2^ = 0.88855; R^2^ adj = 0.81890), the thickness swelling model (R^2^ = 0.81103; R^2^ adj = 0.72704) and the water absorption model (R^2^ = 0.78866; R^2^ adj = 0.65657). The lower adjusted coefficients for the moisture-related responses indicate that water absorption and thickness swelling were more sensitive to local structural variations, such as open porosity, fine-particle accumulation and non-uniform polymer coverage. Accordingly, the models were used mainly for interpreting response trends and identifying rational candidate regions, rather than for externally validated prediction.

The difference between the physical and mechanical models is reasonable. Water absorption and thickness swelling are strongly affected by local defects, open porosity, exposed wood-rich zones and the degree of polymer coverage, which may vary within manually formed laboratory panels produced from a heterogeneous recycled feedstock. Mechanical properties, especially modulus of elasticity and bending strength, are more directly governed by density, consolidation and the continuity of the load-bearing structure. For this reason, the response surfaces are used here mainly as empirical tools for interpretation and screening, while future work should include additional validation boards at the predicted candidate regions.

### 3.6. Water Absorption

The final uncoded regression model for 24 h water absorption was:

*A* = 380.23406 − 5.2759713x_1_ − 0.56434282x_2_ + 0.16448372x_1_^2^ + 0.00026405x_2_^2^ − 0.00167307x_1_x_2_

where A is the water absorption after 24 h immersion (%), x_1_ is the wood fiber content (wt.%), and x_2_ is the target density (kg·m^−3^).

**Figure 4:**
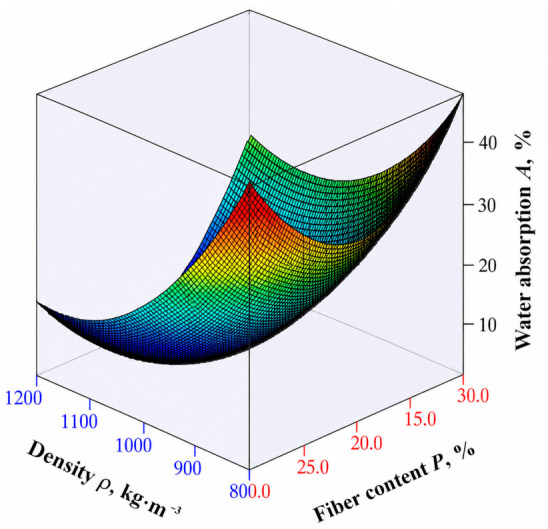
Water absorption of the panels as a function of wood fiber content and panel density.

The combined effect of wood fiber content and panel density on 24 h water absorption is presented in Figure 4.

The response surface indicates that increasing density generally reduced water absorption within the studied range. At lower densities, the structure is more open, and the probability of interparticle voids between recycled ABS particles and insufficient contact between the polymer-rich phase and wood fibers is higher. Such voids and weakly covered regions may act as capillary pathways for water penetration. At higher densities, the reduced void volume and improved furnish packing can limit water access to the internal structure.

This trend is consistent with earlier studies on flat-pressed WPC panels, where higher density and improved compaction were associated with reduced void content, better particle contact and improved physical performance [3,4,6,7]. In the present recycled ABS-based boards, the effect should be interpreted as a combined result of densification and the ability of the ABS-rich phase to consolidate during pressing. Because contact-angle or surface-energy measurements were not performed, the discussion of moisture resistance is based on the measured water absorption and thickness swelling values rather than on direct surface hydrophobicity data.

The influence of wood fiber content was nonlinear. Minimum water absorption was not predicted at the lowest fiber content, which suggests that a moderate fiber share may improve furnish packing or reduce open voids in the pressed structure. At higher fiber contents, however, the relative amount of hydrophilic lignocellulosic material increases, while the available ABS-rich matrix per unit of wood surface decreases. This may lead to partially covered fibers and higher water uptake. Because the predictive ability of the water absorption model was limited, this optimum is treated as a candidate region rather than a validated minimum.

This interpretation is in agreement with studies on ABS/wood and other WPC systems, which have shown that the addition of lignocellulosic particles can improve stiffness but may negatively affect water resistance when the polymer matrix is insufficient to cover the wood phase [19,20,21,22]. In the present study, the water resistance of recycled ABS-based panels is therefore interpreted as a function of density, wood fiber content and visible structural uniformity, while quantitative analysis of matrix coverage remains a subject for future work.

**Figure 5:**
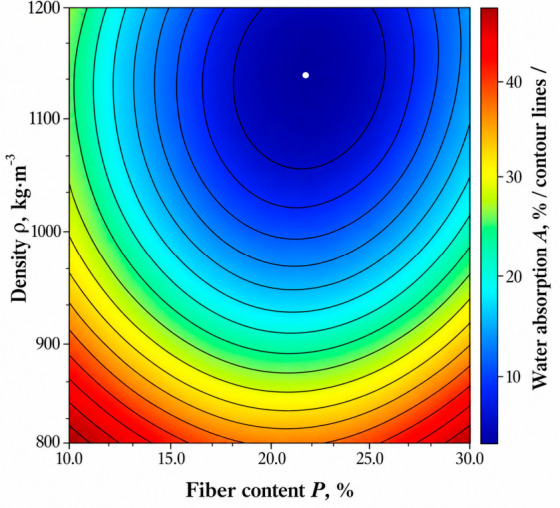
Optimization map for water absorption and localization of the predicted minimum.

As shown in Figure 5, the calculated minimum water absorption was located at an intermediate wood fiber content and an intermediate-to-high panel density. The predicted minimum was obtained at 21.84 wt.% wood fibers and 1134 kg·m^−3^, where the model predicted a water absorption value of 1.62%. At this point, the predicted thickness swelling was 3.22%, the modulus of elasticity was 2931 N·mm^−2^, and the bending strength was 22.20 N·mm^−2^. Because the water absorption model showed limited predictive robustness, this point is discussed as a rational candidate region that combines low water uptake with acceptable mechanical and dimensional-stability indicators, not as a fully validated optimum.

From a technological perspective, this result suggests that low water absorption may be achieved by selecting a balanced fiber-to-ABS ratio and sufficient panel density rather than simply minimizing the wood fiber content. Around 22 wt.% wood fibers, the structure may benefit from improved packing without reaching a fiber content at which the hydrophilic phase dominates the moisture response. Replicated verification boards at this candidate region are required before practical optimization can be claimed.

### 3.7. Thickness Swelling

The final uncoded regression model for 24 h thickness swelling was:

*G_t_* = 133.51484 − 0.69496538x_1_ − 0.23583761x_2_ + 0.01797692x_1_^2^ + 0.00011179x_2_^2^

where G_t_ is the thickness swelling after 24 h immersion (%), x_1_ is the wood fiber content (wt.%), and x_2_ is the target density (kg·m^−3^).

**Figure 6:**
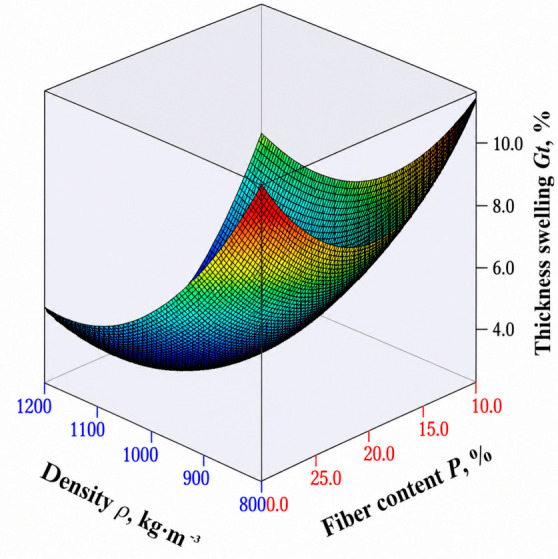
Thickness swelling of the panels as a function of wood fiber content and panel density.

The combined effect of wood fiber content and panel density on 24 h thickness swelling is presented in Figure 6.

The response surface for thickness swelling indicates a minimum at intermediate-to-high densities rather than a purely linear density effect. Initial densification can reduce voids, improve contact between particles and increase the apparent fixation of wood fibers within the consolidated ABS-rich phase. At very high density, however, additional compression may increase stored internal stresses in the wood-containing regions, which can be released during water immersion.

This behavior can be explained by the competition between two mechanisms. The first is the beneficial effect of densification, which reduces porosity and improves structural integrity. The second is compression-set recovery of the lignocellulosic component. During immersion, wood fibers and wood-rich zones absorb moisture and may recover part of their compressed shape, leading to springback and increased thickness swelling. This interpretation is consistent with the measured swelling response, although internal stress and density-profile measurements were not included in the present study.

This interpretation is consistent with previous findings for wood-based panels and WPCs, where thickness swelling is controlled not only by water absorption but also by compression-set recovery, density profile and stability of the compressed lignocellulosic phase [3,4,7,36,38,39]. In WPCs, the polymer matrix can reduce water penetration by partially shielding the wood component; however, it cannot fully prevent swelling when wood-rich zones remain exposed or when internal stresses generated during pressing are released during moisture exposure [34,35,36,37,38,40,41].

**Figure 7:**
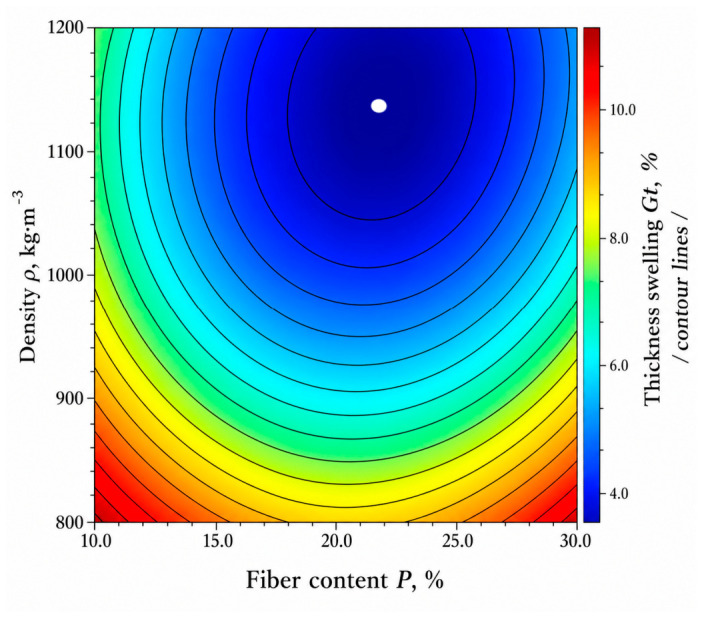
Optimization map for thickness swelling and localization of the predicted minimum.

As illustrated by the optimization map in Figure 7, the predicted minimum thickness swelling was obtained at approximately 19.30 wt.% wood fibers and a density of 1057 kg·m^−3^, with a predicted value of 2.41%. At this point, the predicted modulus of elasticity was 2589 N·mm^−2^, while the bending strength was slightly below 21 N·mm^−2^. Therefore, if a bending strength of at least 21 N·mm^−2^ is required according to the selected EN 622-5-based threshold [42], the optimum for thickness swelling should be shifted slightly towards higher density or towards a composition that improves mechanical resistance without substantially increasing swelling.

The results suggest that thickness swelling is governed more strongly by density and structural consolidation than by fiber content alone. This is important for the design of recycled ABS-based panels, because dimensional stability can be improved through density control and better feedstock preparation, rather than only by reducing the amount of wood fibers.

### 3.8. Modulus of Elasticity

The final uncoded regression model for modulus of elasticity was:

*E_m_* = −6574.7713 − 209.86693x_1_ + 17.102061x_2_ + 0.48962941x_1_^2^ − 0.0083598x_2_^2^ + 0.21047625x_1_x_2_

where *E_m_* is the modulus of elasticity in bending (N·mm^−2^), x_1_ is the wood fiber content (wt.%), and x_2_ is the target density (kg·m^−3^).

**Figure 8:**
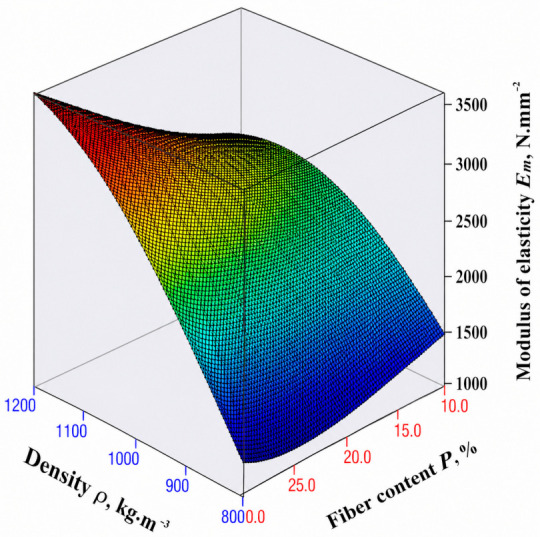
Modulus of elasticity of the panels as a function of wood fiber content and panel density.

The combined effect of wood fiber content and panel density on the modulus of elasticity values of the laboratory-produced panels is presented in Figure 8.

The modulus of elasticity increased strongly with density within the studied range, indicating that densification is a key mechanism responsible for board stiffness. Higher density reduces porosity, increases the number of effective contacts between particles and improves the continuity of the load-bearing structure. This is consistent with previous research on flat-pressed WPC panels, where density and compaction were identified as key factors controlling bending performance [3,4,6,7].

Increasing wood fiber content also contributed to higher stiffness, especially when combined with higher density. This is expected because lignocellulosic fibers are stiffer than the thermoplastic matrix and can act as a reinforcing phase when they are sufficiently incorporated into the consolidated structure. Similar effects have been reported for ABS/wood flour and ABS/wood sawdust composites, where the wood component increased stiffness but the final performance depended strongly on dispersion and interfacial quality [14,19,20,21,22,34,35,36,37,40,41].

The combined effect of density and fiber content indicates that the reinforcing role of the wood fibers becomes more pronounced when the composite is sufficiently compacted. At lower densities, the same fiber content may be less effective because of voids, weak contact zones and incomplete polymer coverage. This explanation is based on response-surface trends and optical surface observations; direct verification of stress transfer would require fracture-surface analysis and mechanical micromechanical testing.

The response surface also suggests that the effect of density tends to approach a plateau at high density levels. Beyond a certain degree of consolidation, further densification may not lead to a proportional increase in stiffness because local heterogeneity, imperfect fiber incorporation, residual stresses or limited polymer redistribution may become controlling factors.

**Figure 9:**
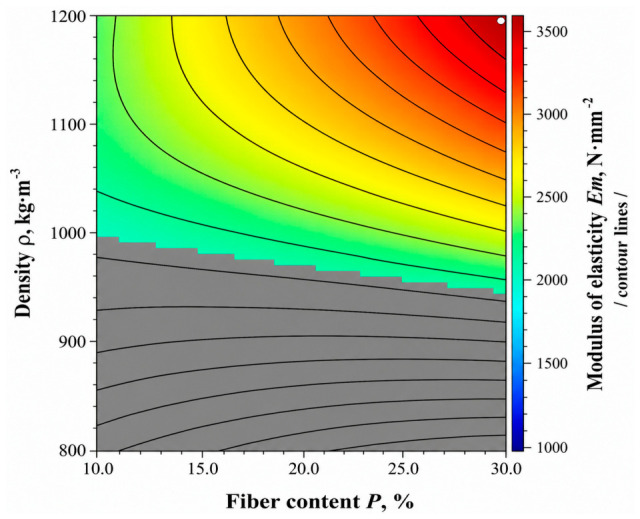
Optimization map for modulus of elasticity and localization of the predicted maximum.

As illustrated in Figure 9, the predicted maximum modulus of elasticity was obtained at 29.97 wt.% wood fibers and a density of 1191 kg·m^−3^, where the model predicted 3598 N·mm^−2^. At this point, the predicted bending strength was 28.18 N·mm^−2^ and the thickness swelling was 6.52%, indicating high mechanical performance and acceptable dimensional stability within the investigated range. However, the predicted water absorption was 12.55%, showing that maximum stiffness is achieved at the expense of reduced moisture resistance.

This trade-off is typical for wood–polymer composites. Increasing the lignocellulosic fraction improves stiffness, but also increases the amount of hydrophilic material and the number of fiber–matrix interfaces that may become water pathways if not fully encapsulated [17,18,19,20,21,22].

### 3.9. Bending Strength

The final uncoded regression model for bending strength was:

*fm* = −110.65255 − 2.7776863x_1_ + 0.27423922x_2_ + 0.03730882x_1_^2^ − 0.00013085x_2_^2^ + 0.0013325x_1_x_2_

where *fm* is the bending strength (N·mm^−2^), x_1_ is the wood fiber content (wt.%), and x_2_ is the target density (kg·m^−3^).

**Figure 10:**
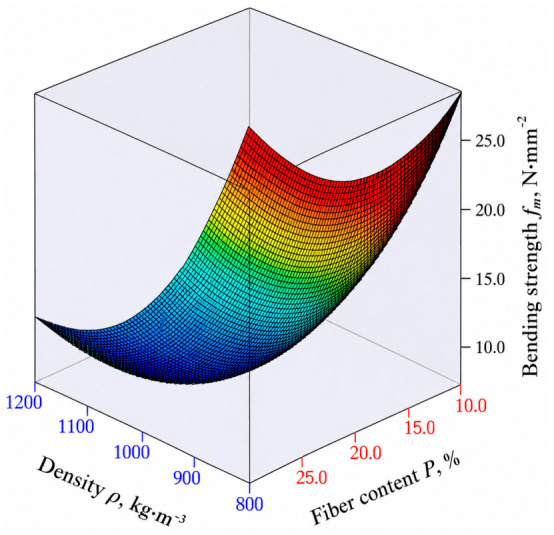
Bending strength of the panels as a function of wood fiber content and panel density.

A graphical representation of the combined effect of wood fiber content and panel density on the bending strength values of the laboratory-produced panels is presented in Figure 10.

As with the modulus of elasticity, panel density was the most influential factor controlling bending strength. Bending strength is highly sensitive to voids, weak interparticle contacts, incomplete matrix consolidation and local structural defects. At higher densities, the reduced void volume and improved furnish packing enhance contact between recycled ABS particles and wood fibers, while the softened ABS-rich phase can form a more continuous load-bearing structure. As a result, internal discontinuities are reduced and the effective stress-bearing cross-section of the panel is increased.

The influence of wood fiber content was nonlinear and depended on density. Wood fibers can contribute to bending strength when the composite structure is sufficiently compacted and the ABS-rich phase provides adequate incorporation of the fibers. Under high-density conditions, closer contact between the softened ABS-rich phase and wood fibers may improve load transfer, allowing the lignocellulosic phase to contribute more effectively to load bearing. At lower densities, however, the same fibers may act as stress concentrators or weak points if they are poorly incorporated or located in regions with limited polymer coverage.

This behavior is in agreement with previous studies on ABS/wood composites, where the presence of wood particles or fibers increased stiffness but did not always lead to proportional improvements in strength. Strength development in such systems depends on matrix continuity, dispersion quality, interfacial interactions and the presence of voids or agglomerates [14,19,20,21,22,34,35,36,37,40,41]. In the present work, these factors are inferred from macroscopic testing and optical surface observations; therefore, statements concerning interfacial adhesion or stress transfer are treated as mechanistic interpretations rather than direct experimental proof. Detailed SEM fracture analysis and compatibilization studies will be addressed in future work.

**Figure 11:**
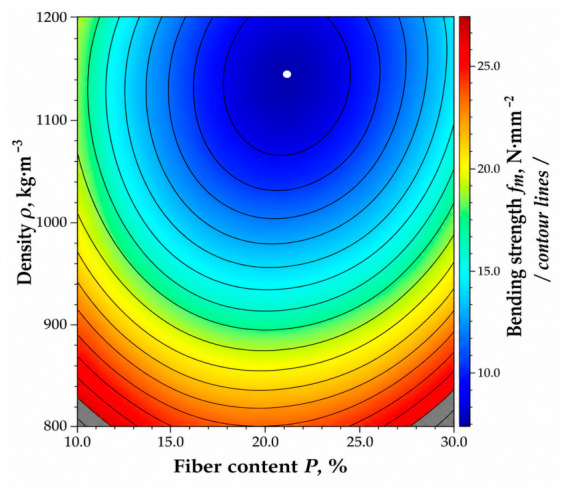
Optimization map for bending strength and localization of the predicted maximum.

As illustrated in Figure 11, the predicted maximum bending strength was obtained at 29.88 wt.% wood fibers and a density of 1196 kg·m^−3^, with a predicted value of 28.09 N·mm^−2^. At this point, the predicted modulus of elasticity was 3609 N·mm^−2^, thickness swelling was 6.64%, and water absorption was 12.40%. Therefore, the maximum strength region almost coincides with the maximum stiffness region. This indicates that, within the investigated factor space, the mechanical candidate region is located at high density and high fiber content.

However, this mechanical candidate region is not identical to the region predicted for minimum water absorption. The higher fiber content improves stiffness and strength but also increases the hydrophilic component of the composite and the number of fiber-matrix interfaces. Therefore, for applications where moisture resistance is critical, the mechanical candidate region may not be the most appropriate technological solution. This compromise between stiffness, strength and moisture resistance is consistent with broader WPC literature [36,37,40,41].

### 3.10. Combined Evaluation of the Properties and Selection of Rational Factor Levels

The combined analysis of the four response surfaces shows that panel density is the main factor that opens the region of acceptable performance. For orientation, the selected performance limits for thickness swelling, modulus of elasticity and bending strength were compared with the relevant MDF requirements in EN 622-5 [42]. At densities below approximately 950–1000 kg·m^−3^, the risk of insufficient bending strength and modulus of elasticity is high. At densities above approximately 1100 kg·m^−3^, the mechanical requirements are satisfied more consistently over a wider range of fiber contents. However, the swelling response shows that excessively high density is not always optimal for dimensional stability because of possible stress relaxation and springback during water immersion.

The most balanced candidate solution, when low water absorption is combined with acceptable mechanical performance, was predicted at 21.84 wt.% wood fibers and 1134 kg·m^−3^ density. At this point, the predicted values were 1.62% water absorption, 3.22% thickness swelling, 2931 N·mm^−2^ modulus of elasticity and 22.20 N·mm^−2^ bending strength. Because the water absorption model showed limited predictive robustness, this point should be understood as a rational region for further verification, not as a fully validated optimum.

If the priority is minimum thickness swelling, while maintaining a modulus of elasticity above 2200 N·mm^−2^ and bending strength above 21 N·mm^−2^ [42], a rational compromise is located close to 20–21 wt.% wood fibers and approximately 1070–1080 kg·m^−3^ density. In this region, thickness swelling remains close to its predicted minimum, while bending strength reaches the selected threshold and modulus of elasticity remains above the selected limit.

If maximum mechanical performance is required, the best candidate region is close to 30 wt.% wood fibers and 1190–1200 kg·m^−3^ density. This region provides predicted modulus of elasticity values around 3600 N·mm^−2^ and bending strength around 28 N·mm^−2^, while thickness swelling remains below 10%. However, water absorption increases to approximately 12–13%, which indicates that additional technological improvements would be needed if high mechanical performance and low water uptake are required simultaneously.

Overall, the results confirm that recycled ABS edge-banding waste can function as a thermoplastic matrix for producing compact wood–polymer composite boards. The decisive technological condition is not only the nominal ABS-to-fiber ratio, but also the quality of the recycled ABS fraction. Sieving and removal of excessive dust-like material after grinding are expected to improve particle-size uniformity and reduce local fine-rich zones. The magnitude of these improvements should be quantified in subsequent studies focused specifically on feedstock preparation and morphology–property relationships.

### 3.11. Scope and Limitations of the Structure–Property Interpretation

This subsection defines the scope of the structure–property interpretation presented in this work. The relationships discussed in the manuscript are based on formulation variables, panel density, fractional composition of the recycled ABS fraction, qualitative optical surface microscopy and the measured physical and mechanical properties. Therefore, they should be regarded as empirical structure–property relationships within the investigated factor space rather than as a complete mechanistic description of the recycled ABS–wood fiber system.

The chemical composition of the recycled ABS stream, the exact quantity and nature of the fine fraction, interfacial adhesion, surface energy, thermal transitions and microscale stress-transfer mechanisms were not directly determined in the present study. Accordingly, interpretations related to fine residues, apparent matrix coverage, fiber incorporation, water-ingress pathways and load transfer are presented as plausible mechanisms supported by the experimental trends, optical observations and the literature, but not as independently quantified effects. These aspects will be addressed in subsequent studies focused on feedstock characterization, thermal-processing behavior, detailed morphology and long-term performance of recycled ABS-based wood–polymer composite boards.

## 4. Conclusions

This study demonstrated the feasibility of producing hot-pressed wood–polymer composite boards using recycled ABS edge-banding waste as a thermoplastic matrix and industrial wood fibers as the lignocellulosic phase. The main contribution is the evaluation of a real furniture-industry ABS waste stream in denser panel-type composites produced without added formaldehyde-based resins.

Panel density was the dominant factor governing board performance. Higher density generally improved modulus of elasticity and bending strength and reduced water absorption, whereas the most balanced predicted region for combined moisture resistance and mechanical performance was located at approximately 22 wt.% wood fibers and 1130 kg·m^−3^. The calculated candidate point at 21.84 wt.% wood fibers and 1134 kg·m^−3^ gave predicted values of 1.62% water absorption, 3.22% thickness swelling, 2931 N·mm^−2^ modulus of elasticity and 22.20 N·mm^−2^ bending strength.

The regression models were statistically significant and useful for interpreting the main trends within the investigated factor space. However, the predicted optima should be regarded as candidate regions rather than externally validated processing conditions.

Qualitative optical microscopy indicated that more homogeneous surfaces were associated with a more continuous ABS-rich phase, while local accumulations of fine particles and areas of limited polymer coverage were visible on some surfaces. These observations support the practical recommendation that ground ABS edge-banding waste should be additionally sieved or cleaned before board manufacture in order to improve particle-size uniformity and reduce local fine-rich zones.

The main limitations of this study are its laboratory scale, the use of one recycled ABS waste source and the restricted range of fiber contents, densities and pressing conditions. Therefore, the predicted candidate regions and the proposed structure–property interpretations should be verified in future work using additional recycled feedstocks, replicated boards at the selected factor levels, and complementary chemical, thermal and morphological analyses.

## Figures and Tables

**Table 1:** Experimental design for laboratory board production.

Target ρ, kg m^−3^	ABS Waste, wt.%	Wood Fibers, wt.%	X_2_	X_1_	Panel
800	90	10	−	−	1
1200	90	10	+	−	2
800	70	30	−	+	3
1200	70	30	+	+	4
1000	90	10	0	−	5
1000	70	30	0	+	6
800	80	20	−	0	7
1200	80	20	+	0	8
1000	80	20	0	0	9

**Table 2:** Variation statistics for the density of the laboratory panels (*n* = 6).

Dev., %	P, %	SE, kg m^−3^	CV, %	SD, kg m^−3^	Mean ρ, kg m^−3^	Target ρ, kg m^−3^	Panel
−0.04	2.32	18.5	5.68	45.4	799.7	800	1
−3.06	0.90	10.5	2.22	25.8	1163.3	1200	2
−3.08	1.50	11.6	3.68	28.5	775.4	800	3
+2.64	0.31	3.8	0.76	9.3	1231.7	1200	4
+1.45	1.37	13.9	3.37	34.2	1014.5	1000	5
−1.16	1.38	13.6	3.37	33.3	988.4	1000	6
−2.61	2.44	19.0	5.97	46.5	779.1	800	7
+0.71	0.69	8.3	1.69	20.4	1208.5	1200	8
−1.25	0.80	7.9	1.95	19.3	987.5	1000	9

**Table 3:** Statistical indicators of the regression models.

Response	Model *p*-Value	R^2^	R^2^ Adj	Residual SE
Water absorption	0.01364	0.78866	0.65657	10.189
Thickness swelling	0.00258	0.81103	0.72704	1.897
Modulus of elasticity	0.00121	0.88855	0.81890	315.75
Bending strength	0.00041	0.91591	0.86335	2.357

## Data Availability

The original contributions presented in this study are included in the article. Further inquiries can be directed to the corresponding author.
